# Enhanced Follicular Delivery of Finasteride to Human Scalp Skin Using Heat and Chemical Penetration Enhancers

**DOI:** 10.1007/s11095-020-02822-y

**Published:** 2020-05-31

**Authors:** H.A. Farah, M.B. Brown, W.J. McAuley

**Affiliations:** 1 Centre for Research in Topical Drug Delivery and Toxicology, University of Hertfordshire, College Lane, Hatfield, AL10 9AB UK; 2 MedPharm Ltd, Unit 3 Chancellor Court, 50 Occam Road, Surrey Research Park, Guildford, GU2 7AB UK

**Keywords:** androgenetic alopecia, Chemical penetration enhancers, finasteride, follicular drug delivery, heat, skin permeation, topical drug delivery

## Abstract

**Purpose:**

The aim of this work was to evaluate whether improved topical delivery of finasteride, focussed to the hair follicles of human scalp skin could be achieved with application of short durations of heat and use of specific chemical penetration enhancers.

**Methods:**

Franz cell experiments with human scalp skin were performed with a range of chemical penetration enhancers at 32°C and 45°C to simulate normal and heated conditions. Selected chemical penetration enhancers were taken forward for finite dose Franz cell studies which examined the effect of heat produced by a prototype external heating system that supplied either 20 or 30 min of additional heat over both a 24 h and a 1 h time period.

**Results:**

Short durations of externally applied heat significantly increased finasteride penetration into human scalp skin after 24 h. Analysis of drug distribution in the skin after 1 h and 24 h indicated that both heat and chemical penetration enhancer selection influenced drug delivery to the hair follicles.

**Conclusion:**

The use of short durations of heat in combination with specific chemical penetration enhancers was able to increase the delivery of finasteride to human scalp skin and provide focussed drug delivery to the hair follicles.

## Introduction

Finasteride is used systemically as an oral medication for the treatment of androgenetic alopecia (AGA) in men, which is commonly known as male pattern baldness. Finasteride inhibits the type II isoenzyme of 5-alpha reductase present in hair follicles, decreasing the local level of dihydrotestosterone (1). In scalps with AGA, dihydrotestosterone causes progressive miniaturisation of the hair follicles and shortening of the different phases of the hair growth cycle producing hair loss (2,3). Finasteride reverses these processes, preventing further hair loss and induces hair regrowth. Systemic administration of finasteride causes several undesirable side-effects, including reduced libido and impairment of spermatogenesis in men (4,5). Moreover, finasteride is not recommended for use in women because of the risk of feminising male foetuses during pregnancy. However because of a lack of other suitable treatments, it is used off label for the treatment of hirsutism and AGA in women (6–9). If effective concentrations of finasteride could be delivered by topical administration to the hair follicles, treatment could be provided, and the side effects associated with systemic delivery could potentially be minimised or prevented. The high follicular surface area of human scalp skin and that the hair follicles are the drug target site suggests that such an approach would be feasible (10,11). However, features such as the high follicular density of scalp skin are thought to increase drug absorption to a greater extent for hydrophilic drugs in comparison to lipophilic molecules such as finasteride making it difficult to predict how effective follicular delivery of finasteride from topical administration would be (12,13). Recently the use of a heating system that can provide a physiologically tolerable increase in the skin temperature for relatively short periods of time along with selected chemical penetration enhancers has shown promise for delivery of drugs to the hair follicles, including for lipophilic molecules such as isotretinoin (14,15). An understanding of the effects of this enhancement approach on clinically relevant, model skin tissue such as human scalp skin would enable the potential of heat and chemical penetration enhancers to improve the follicular delivery of finasteride to be evaluated. Here the transport of finasteride into and through human scalp skin in the presence of heat and chemical penetration enhancers has been assessed with in vitro Franz diffusion cells. A water bath was used initially to provide heated conditions for the experiments to identify suitable chemical penetration enhancers. Following this a prototype heating system and formulation vehicles were used to investigate the effect of practically feasible, short, durations of heat with chemical penetrations enhancers on drug delivery to different compartments in the skin.

## Materials and Methods

### Materials

Finasteride (99.9%) was purchased from Sequoia Research Products (Pangbourne, UK). Acetonitrile HPLC grade (99.9%), ethanol HPLC grade (EtOH, 99.9%), isopropyl alcohol (IPA, 99.9%), methanol HPLC grade (99.9%), propylene glycol (PG, > 99.0%), phosphate buffer saline (PBS) tablets (10 mM, pH 7.4), Dura Seal™ (Diversified Biotech, USA), Hamilton GASTIGHT® syringes (Hamilton®, Switzerland) and Parafilm M® laboratory film (Bemis® Flexible packaging, USA) were acquired from Fisher Scientific (Loughborough, UK). Sodium thiosulfate pentahydrate (ST, 99.5%) was bought from Acros Organics (New Jersey, USA). Diisopropyl adipate (DPA, > 99.8%) and dimethyl isosorbide (DMI, > 99.8%) were supplied by Croda (Barcelona, Spain). Transcutol® P (TP, > 99.8%) was provided by Gattefosse (France). The 2-octyl cyanoacrylate glue was sourced from Adhezion Biomedical (Wyomissing, USA).

### Quantitative Analysis of Finasteride

Finasteride was assayed using HPLC (Shimadzu Corp., Kyoto, Japan). The system consisted of a pump (LC-20 AD) with a degasser (DGU-20A_5R_), auto-sampler (SIL-20A HT), column oven (CTO-20 AC), UV-VIS detector (SPD-20A) and communication module (CBM-20A). Data acquisition was performed on LabSolutions software® version 5.54 SP2. Chromatographic separation was performed using a Thermo Scientific™ Hypersil™ ODS C18 (5 μm, 250 × 4.6 mm, Thermo Fisher Scientific, Leicestershire, UK) at room temperature. The detection wavelength was 210 nm. The mobile phase consisted of 60% acetonitrile: 40% deionised water at a flow rate of 0.5 mL/min. The run time was 15 min. The injection volume was 10 μL and the retention time for finasteride was 10.3 min. The HPLC methods were validated for linearity, precision and accuracy according to current ICH guidelines and the LOD and LOQ were 0.32μg/mL and 0.97 μg/mL respectively (16).

### Determination of Finasteride Solubility in Donor Vehicles

Solubility studies were conducted by preparing saturated suspensions of finasteride in each solvent (DMI, DPA, IPA, PG and TP) in glass vials at both 32°C and 45°C (these were the skin surface temperatures employed during the in vitro skin permeation studies). A PTFE coated magnetic stirrer bar was introduced into each vial, after which the vials were tightly sealed and covered with Parafilm®. The suspensions were stirred for 24 h at the stated temperatures, after which samples were removed and filtered using 0.2 μm PTFE syringe filters (dot-red® analytical, UK). Where appropriate the filtrate was diluted with methanol prior to analysis.

### Preparation of Donor Formulations/Suspensions

To prepare formulations for the infinite dose in vitro permeation experiments, excess drug was added to the solvent vehicles (DMI, DPA, IPA, PG and TP) and stirred for 24 h in a water bath (Grant Instruments, UK) at the appropriate temperatures (32°C or 45°C) to produce saturated solutions which were used directly. For the finite dose in vitro permeation studies, finasteride saturated solutions were prepared at 32°C and 45°C (which were filtered before use) in 1:1 binary systems of IPA: PG and IPA: TP.

### Skin Preparation

Full thickness human scalp skin from a single male cadaveric donor was obtained from Tissue Solutions (UK) with informed consent and appropriate ethical approval (EC/2012/29/MedPharm). The skin was prepared by carefully trimming the hair using a Gillette Fusion ProGlide Styler® Trimmer. The subcutaneous fat was then carefully removed using a forceps and scalpel and the skin was stored in a freezer at −20°C until it was needed for use.

### Infinite Dose In Vitro Skin Permeation Studies

Skin samples were placed on the individually calibrated unjacketed upright Franz diffusion cells (diameter 1.0 cm^2^, volume 3.0 mL) (Soham Scientific, UK), with the SC facing the donor compartment and the dermis facing the receptor. Both chambers were then wrapped together using Parafilm® (at 37°C) or Dura Seal™ (at 50°C) before being clamped together. The receptor chamber was filled with EtOH: PBS solution (30:70% *v*/v) and stirred with a magnetic bar to ensure adequate mixing (600 rpm). The water bath temperature was maintained at either 37°C or 50°C to keep the skin surface at approximately 32± 1°C and 45± 1°C respectively. Prior to dosing, the Franz cells were equilibrated at the appropriate temperature and then the membrane surface temperature was measured from the donor compartment using a Fisher Scientific Traceable Digital Thermometer with a type-K probe. Air bubbles were removed through the sampling arm by carefully tilting or inverting the diffusion cell and checks for leaks were made at the same time. A saturated suspension (0.5 mL) of finasteride was then introduced into the donor chamber. Following this the receiver fluid (200 μL) was removed from receptor compartment via the sampling arm at regular intervals and analysed via HPLC. An equal volume of pre-warmed receiver fluid was immediately added to replace the sampled volume. Six repetitions (*n* = 6) of each experiment were performed.

### Finite Dose In Vitro Skin Permeation Studies

Filtered saturated solutions of finasteride in IPA: PG (1:1) and IPA: TP (1:1) were prepared at 32°C and 45°C and used to dose Franz cells mounted with human scalp skin with finite doses of 10 μL cm^−2^. As before the Franz cells had been pre-equilibrated in water baths maintained at either 37°C or 50°C to keep the skin surface at approximately 32± 1°C and 45± 1°C respectively. Additionally, the effect of controlled local heating on drug permeation across skin was determined as described previously (15). To summarise, after applying the formulation prepared at 32°C to the donor chamber, 2.5 mL or 5 mL of Sodium thiosulfate pentahydrate (ST) solution was introduced into the donor chamber to produce a membrane surface temperature of ~ 43°C - 44°C for approximately 20 and 30 min respectively. Aluminium foil was used to separate the heating system from the formulations. For experiments with no ST solution, utilising only a water bath (37°C & 50°C), aluminium foil was also placed in the donor chamber of each Franz cell to mimic the experiments using the ST chemical heating system. These studies were conducted over 24 h and 1 h. Six repetitions (*n* = 6) and four repetitions (*n* = 4) were performed for each treatment group for the 24 h and 1 h duration experiments respectively. All other parameters, and procedures were consistent with those described for the infinite dosing experiments.

### Determination of Drug Content in Hair Follicles and Skin Tissue

After the completion of the infinite dose diffusion experiments, the skin was removed from the Franz cells and carefully dried by patting with tissue paper. The residual formulation was removed with three separate cleaning phases. The first cleaning phase was conducted by carefully rolling a dry cotton bud over the skin upwards three times, then downwards three times, then clockwise and anticlockwise along the edges once. For the second cleaning phase, the first cleaning phase was repeated using a wet cotton bud soaked in methanol. For the third cleaning phase, the first cleaning step was repeated using a dry cotton bud. All three buds were then discarded. To remove any remaining surface formulation two tape strips (Scotch® Tape strips, 3 M Center, USA) were taken and discarded. To remove the SC, a further ten tape strips were taken and placed into an amber glass vial. Following SC removal, the skin was placed in a benchtop oven (Binder GmbH, Germany) at 60°C for 2 min which allowed the epidermis to be separated from the dermis more easily. Epidermal and dermal samples were transferred into individual glass vials. Then methanol (2.0 mL) was added to each vial, which was then sonicated for 20 min before being transferred to a Stuart roller mixer SRT9 (Cole-Parmer, UK) overnight (16 h - 18 h). All samples were then filtered using 0.2 μm PTFE syringe filter (dot-red® analytical, UK) before being analysed by HPLC.

For the finite dose experiments the same process was followed, with the exception that tissue paper was not used to remove excess formulation from the skin surface and the cotton buds used to remove the residual formulation from the aluminium foil (used to separate the ST heating system from the formulation), donor chamber and skin surface were not discarded. To remove any remaining skin surface formulation two tape strips were taken. To determine drug uptake into the hair follicles, differential tape stripping was performed (14) at the end of the finite dose studies. Briefly, 10 tape strips were taken to remove the SC and transferred into a glass vial. After the SC removal, a drop of 2-octyl cyanoacrylate glue was applied onto the tape stripped skin and was covered with a glass slide under slight pressure. After 5 min, the cyanoacrylate superglue polymerized, and the glass slide was removed with one quick movement taking the follicular casts with the slide. The cast was transferred into a glass vial using forceps. The remaining skin was heat separated as described for the infinite dosing experiments. The extraction procedure was repeated for each stage until the drug content in the extracted samples was no longer detectable or below the LOQ and considered fit for purpose. For all of the finite dose experiments, the level of finasteride recovery was over 90% in accordance with OECD guidelines (17).

### Analysis of Permeation Parameters

To provide a mechanistic insight into the role of heat and CPEs in enhancing percutaneous absorption, the experimental permeation data was modelled using Fick’s first law Eq. (1).

1 $$ J=\frac{D\times K\times {C}_V}{h} $$

Where *J* is steady state flux, *D* the diffusion coefficient of the solute, *h* the diffusional path length, *K* the partition coefficient and *C*_*V*_ is the drug concentration in the vehicle. According to Eq. (1), *J* is directly proportional to the partition (*K*) and diffusion (*D*) coefficient of the solute under steady state conditions. Since *J* and the lag time (*T*_*L*_) can be readily determined from the diffusion profile (plot of the cumulative amount permeated per unit area (Q) vs time (t)), as the gradient and x-intercept of the linear portion of the graph (18 h – 24 h) respectively, *D* and *K* can theoretically be obtained. However, since the diffusional pathlength across the SC (*h*) is unknown, these cannot be calculated directly, but can be calculated as pathlength normalised values (*D*/*h*^2^ and *Kh*) as illustrated in Eq. (2) and Eq. (3) (18).

2 $$ \frac{D}{h^2}=\frac{1}{6\times {T}_L} $$

3 $$ K\times \kern0.5em h\kern0.5em =6\kern0.5em \times \left(\ \frac{\mathrm{J}}{{\mathrm{C}}_{\mathrm{V}}}\kern0.5em \right)\times {T}_L $$

4 $$ K\times h=6\times \left(\ \frac{J}{\upalpha\ }\ \right)\times {T}_L $$

The use of *C*_*V*_ in calculating *Kh* was expected to lead to under estimation of the *Kh* values because of increased finasteride solubility in the vehicles at the higher temperature. The use of *C*_*V*_ in Fick’s law is common as it is convenient to measure, however it is commonly recognised that it is the thermodynamic activity of the drug in the vehicle that fully accounts for the diffusional gradient across the skin, particularly when comparing between different formulations or conditions (14,19). Therefore, the thermodynamic activity (α) of finasteride in the vehicles, which as the formulations were saturated solutions has a value of unity, to model the experimental data obtained at both temperatures (32°C and 45°C). Thus, *C*_*V*_ in Eq. (3) was replaced with the thermodynamic activity (α) to give Eq. (4).

### Statistical Analysis

Statistical analysis of all the data was performed using GraphPad Prism version 8.00 for Windows, (GraphPad Software, La Jolla California, USA). The initial screening of the effect of temperature and the full range of penetration enhancers was performed using a two-way ANOVA. Subsequent statistical comparisons were then performed using a one-way ANOVA for parametric data or Kruskal-Wallis test for non-parametric data. Post hoc comparisons were made with either Fishers LSD test, Tukey’s HSD test or Dunn’s for parametric and non-parametric data as appropriate. Similarly, pair wise comparisons were made with either a *t*-test or Mann Whitney *U* test as appropriate. Statistically significant differences were assumed at the 95% confidence level, i.e., when *p* < 0.05.

The enhancement ratios (*E*_*R*_) were determined using Eq. (5) below:

5 $$ {E}_R=\frac{Q(E)}{Q(C)} $$

where Q (E) and Q (C) are the amount of the drug penetrated into and permeated across the skin when using enhancement strategies (i.e. heat and CPEs) and control (no additional heat and CPEs) respectively. The permeation parameters obtained at different temperature (*J*, *D/h*^*2*^, *Kh* and *T*_*L*_) were then compared to the control (32°C) using the student t-test or Mann-Whitney U test (when data were found to be not normally distributed). Statistically significant differences were accepted at 95% confidence level, i.e., when *p* < 0.05.

## Results and Discussion

### Finasteride Solubility in Donor Vehicles

The saturated solubilities of finasteride (at 32°C and 45°C) in a range of solvents commonly used in topical/transdermal drug delivery formulations that have demonstrated skin penetration enhancing effects was determined and are shown in Table 1 (14,20). The solvents selected included those from different categories of enhancer, e.g. alcohol and fatty acid ester and may therefore work differently with heat and be able to demonstrate synergistic effects on finasteride transport into the skin when used as solvent mixtures (20). Finasteride solubility was highest in IPA, with good solubility observed in all the solvents tested. At the higher temperature of 45°C, finasteride solubility was increased in each solvent; the magnitude of this increase was proportionally similar and ranged between approximately 1.4 and 1.5 fold.

**Table 1:** Saturated solubility of finasteride in various vehicles over 24 h at 32°C and 45°C (the temperatures employed in the skin permeation studies). Mean ± SD (*n* = 3)

Vehicle	Saturated solubility of finasteride (mg/mL)	
	32°C	45°C
DMI	26.21 ± 0.42	39.50 ± 0.81
DPA	6.22 ± 0.51	8.66 ± 0.20
IPA	83.78 ± 1.60	120.15 ± 2.45
PG	49.71 ± 1.02	76.88 ± 1.71
TP	63.23 ± 0.68	91.93 ± 1.05

### Infinite Dose In Vitro Skin Permeation and Distribution Studies

#### Infinite Dose In Vitro Skin Distribution Studies

**Fig. 1:**
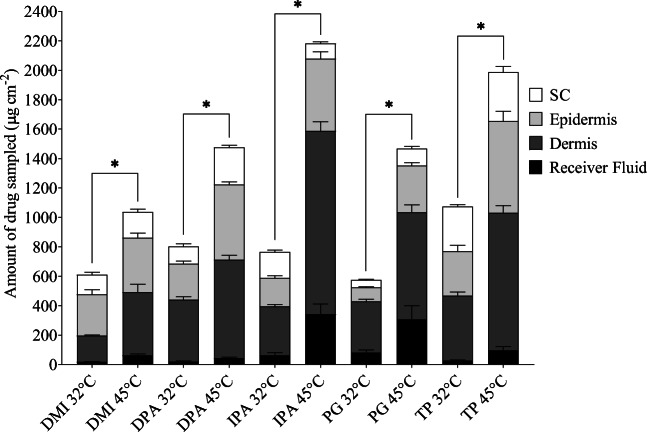
The total amount of finasteride recovered from the *stratum corneum* (SC), epidermis, dermis and receiver fluid at the end of the infinite dose permeation study at 32°C and at 45°C from dimethyl isosorbide (DMI), diisopropyl adipate (DPA), isopropyl alcohol (IPA), propylene glycol (PG) and Transcutol® (TP) vehicles. * indicates significant differences in total finasteride delivery at 45°C. All data bars show the mean + SEM of *n* = 6 diffusion cells

Infinite dose Franz cell studies using human scalp skin were conducted using saturated solutions of finasteride in each of the vehicles above. The total amount of finasteride recovered from the skin tissue; that is the sum of drug recovered from the SC, epidermis and dermis and receiver fluid, as well as the amount in each of these compartments after 24 h at both 32°C and 45°C are shown in Fig. 1. Both the temperature reached and the duration that the skin is held at a particular temperature are known to affect when discomfort or damage is caused to the skin (21–23). The higher temperature of 45°C was used here as it is believed to be physiologically tolerable and suitably high to enable the effects of heat on drug transport into and across skin to be easily elucidated (24,25).

A two-way ANOVA revealed the effects of both vehicle and heat on finasteride delivery were statistically significant (*p* < 0.05). At the higher temperature (45°C), the total amount of finasteride delivered was significantly increased from all vehicles. The magnitude of these increases ranged between 1.7 and 2.9 fold with DMI and IPA respectively producing the smallest and largest enhancements with heat. These solvents also provided lowest and highest total delivery respectively at the higher temperature. At 32°C, TP provided the highest total finasteride delivery which was significantly greater, (between 1.34 and 1.87 fold) than that from all the other vehicles investigated (p < 0.05), with the lowest delivery from PG. Finasteride was saturated and therefore at equivalent thermodynamic activity in the different solvents at both temperatures. The differences in finasteride permeation at 32°C from the different solvents and the relative changes in permeation from them at the elevated temperature indicates that the solvents acted as chemical penetration enhancers and differ in their ability to work with heat to improve finasteride permeation into the skin (14). For each solvent at 32°C, finasteride recovery from the receiver fluid was relatively low in comparison to that of the skin tissue. In most cases the largest amounts of drug were extracted from the dermis though DMI provided a greater level of drug delivery to the epidermis. The overall magnitude of the increases in finasteride total delivery with heat are similar to those obtained for isotretinoin from a range of vehicles with heat across abdominal skin (15). However the vehicles performed differently with the different drugs, for example heat did not significantly increase isotretinoin delivery from PG, whereas finasteride delivery to scalp skin was increased 2.6 fold.

#### Infinite Dose In Vitro Skin Permeation Studies

**Fig. 2:**
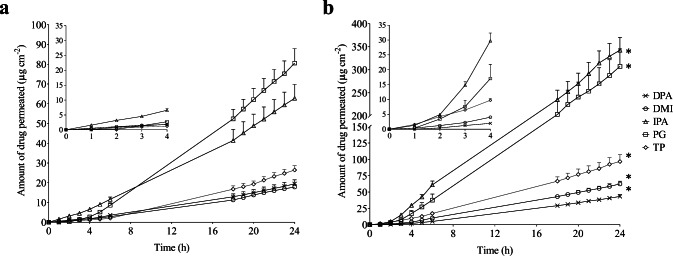
Mean cumulative amount of finasteride permeated through human scalp skin over 24 h: (**a**) at 32°C; (**b**) at 45°C. The inset graphs show an expanded image of the delivery between 0 to 4 h**.** Finasteride was applied (0.5 mL cm^−2^) to the skin surface in a previously equilibrated saturated suspension for each vehicle. Vehicles tested were dimethyl isosorbide (DMI), diisopropyl adipate (DPA), isopropyl alcohol (IPA), propylene glycol (PG) and Transcutol P® (TP). * indicates significant differences in finasteride delivery at 45°C. All points are mean + SEM of n = 6 diffusion cells

To help elucidate the mechanisms through which heat improved finasteride uptake into the skin, the finasteride permeation profiles into the receiver fluid over 24 h from the distribution experiments above were plotted. The plots at 32°C and 45°C show typical infinite dose permeation profiles with an initial lag time followed by a linear permeation profile as shown in Fig. 2(a) and (b) respectively.

At 32°C finasteride was detected in the receiver fluid at early time points (from 1 h onwards) for all the vehicles investigated, with IPA delivering the highest amount over the first six hours. Relatively rapid permeation of drugs across scalp skin is expected in comparison to that of other body sites such as abdominal skin (12). The cumulative amount of finasteride permeated at 24 h ranged from 17.82 ± 1.36 μg/cm^2^ to 80.58 ± 7.30 μg/cm^2^, with DMI and PG producing the lowest and highest receiver fluid concentrations respectively. At 24 h finasteride permeation from PG and IPA was statistically the same and delivery from these vehicles was significantly greater than that from DPA, DMI and TP (*p* < 0.05).

At the higher temperature, increased drug delivery into the receiver fluid from all the vehicles investigated was observed. The increase in receiver fluid concentrations with heat were all statistically significant with DPA and IPA producing the lowest and highest enhancement and the lowest and highest cumulative amounts of drug delivered to the receiver at 24 h respectively with values ranging from 43.03 ± 3.00 μg/cm^2^ to 342.62 ± 27.84 μg/cm^2^. As was observed at 32°C, the amounts of drug delivered to the receiver fluid at 24 h, from IPA and PG were statistically the same and delivery from both was significantly greater than that from TP, DPA and DMI (*p* < 0.05).

To gain a mechanistic understanding of how heat influenced the transport of finasteride across skin when delivered from the various vehicles investigated, Fick’s first law was used to model the linear portion of the permeation data between 18 and 24 h. Although skin permeation is complex, with drug transport occurring potentially via different pathways across the skin, the use of Fick’s law which assumes a single pathway, has been found to be useful for interpreting skin permeation data (26). The pathlength normalised values of partition (*Kh*) and diffusion coefficients (*D*/*h*^2^) were determined and are shown in Table 2. For this analysis, the thermodynamic activity of the drug in each of the solvents was used (α = 1) rather than the concentration of the drug in the vehicle. Ideally to use Fick’s first law, steady state flux would be assessed from a point on the plot 2.7 times past the lag time (27). For some vehicles, this was not possible, however an estimated steady state flux value or pseudo steady state was taken from the linear portion of the plots (18–24 h) as they can still provide mechanistic insight into how heat increase drug permeation across the skin (28).

**Table 2:** Skin permeation parameters measured for finasteride in different dermatological formulations. Cumulative amount permeated (Q_24_), flux between 18 and 24 h ( *J*_18 − 24_), lag time (*T*_*L*_), pathlength normalized partition coefficient (*Kh*), pathlength normalized diffusion coefficient (*D*/*h*^2^) were measured for both physiological skin temperature (32°C) and elevated skin temperature (45°C) using Fick’s first law (Mean ± SEM, n = 6)

Vehicle	Membrane temperature (°C)	*J*_18 − 24_ (μg/cm^2^/h)	E_R_	Q_24_ (μg/cm^2^)	E_R_	*T*_*L*_ (hr)	*D*/*h*^2^(×10^−2^ h^−1^)	E_R_	*Kh* (cm)	E_R_
DPA	32	1.089 ± 0.01	2.16	19.48 ± 1.97	2.20	6.99 ± 0.38	2.38 ± 0.44	1.00	45.67 ± 2.48	2.16
	45	2.352 ± 0.13*		43.30 ± 3.00*		7.00 ± 0.37	2.38 ± 0.45		98.78 ± 5.22*	
DMI	32	1.098 ± 0.06	2.90	17.82 ± 1.36	3.50	6.60 ± 0.45	2.52 ± 0.37	1.46	43.48 ± 2.96	1.98
	45	3.184 ± 0.26*		62.43 ± 4.25*		4.52 ± 0.60*	3.69 ± 0.28*		86.35 ± 11.46*	
IPA	32	3.555 ± 0.28	5.67	62.75 ± 7.04	5.46	6.31 ± 0.49	2.64 ± 0.34	1.02	177.68 ± 10.45	5.56
	45	20.138 ± 1.57*		342.62 ± 27.84*		6.18 ± 0.30	2.70 ± 0.56		746.72 ± 36.25*	
PG	32	4.626 ± 0.41	3.61	80.58 ± 7.30	3.82	6.63 ± 0.56	2.51 ± 0.30	1.06	184.02 ± 15.54	3.42
	45	16.696 ± 1.50*		307.42 ± 37.61*		6.29 ± 0.30	2.65 ± 0.56		630.11 ± 30.54*	
TP	32	1.656 ± 0.11	3.04	26.46 ± 2.15	3.66	7.25 ± 0.49	2.30 ± 0.34	1.73	72.04 ± 4.87	1.75
	45	5.028 ± 0.74*		96.77 ± 10.44*		4.20 ± 0.53*	3.97 ± 0.31*		126.71 ± 15.99*	

*denotes significant difference in the parameter at 45°C for each vehicle compared to the same respective vehicle at 32°C

The flux between 18 and 24 h (*J*_18–24_), was significantly greater for all solvent systems at the higher temperature (2.16 to 5.67 fold). For TP, the enhancement in flux was equally attributed to improvements in *Kh* (1.73 fold) and *D*/*h*^2^ (1.75 fold). For all the other vehicles investigated the increase in flux could primarily be attributed to improvements in *Kh* which was significantly increased for all vehicles, with the enhancement being up to 5.56 fold with IPA. In contrast, the increase in temperature to 45°C had smaller effects on *D*/*h*^2^ produced from these vehicles which were only statistically significant (*p* < 0.05) for DMI (1.46 fold). The effects of heat improving drug delivery across skin primarily through improvements in *Kh* were seen previously for the delivery of isotretinoin across abdominal skin, however the enhancement magnitudes were larger. For example with DPA as the vehicle, heat increased *Kh* 13.6 fold for isotretinoin across human abdominal skin but only 2.2 fold here for finasteride across human scalp skin (15). Heat is thought to potentially have a number of effects on the skin permeation process for example increasing drug diffusivity in the formulation vehicle and skin, affecting sebum viscosity and the packing of the intercellular stratum corneum lipids which may influence drug diffusion through and partitioning into the skin (24,25,29–31). That the increased skin permeation in response to heat was largely attributable to improved drug partitioning into the skin helps explain why enhancers such as propylene glycol appear to behave differently with heat for different drugs, for example providing no significant improvement in isotretinoin skin permeation (15) but significantly the improving skin permeation of finasteride with heat here. Chemical penetration enhancers would be expected to have different effects on the partitioning of drugs with different physiochemical properties into the skin. Isotretinoin which has a log P of 6.6 is considerably more lipophilic than finasteride which has a log P of 3.2 (32,33). Other work that investigated the penetration of erythromycin (log P 2.54) (34) into the skin under the influence of heat and CPEs found that similar to here, more polar CPEs such as TP provided larger increases in skin permeation with heat in comparison to the more lipophilic, fatty acid ester solvent DPA (14). In contrast isotretinoin penetration was increased to the largest extent by propylene glycol monolaurate, another fatty acid ester in combination with heat with a smaller increase provided by the more polar TP and no significant increase provided by PG. This suggests that heat will work more effectively with more lipophilic enhancers for the delivery of highly lipophilic drugs and with more polar enhancers for drugs such as finasteride and erythromycin which have log *P* values close to those considered optimal for delivery across skin. However, care must be taken with this interpretation as these data were generated using skin from different anatomical areas. Human scalp skin which was used in this study has distinctive characteristics and has been shown to be more permeable to substances in comparison to e.g. abdominal skin that was used in the isotretinoin work. In particular the hair follicle density has been reported to be up to twelve times that of abdominal skin with the mean diameter of terminal follicles being twice that of abdominal skin (11). In contrast the number of cell layers making up the stratum corneum and the stratum corneum thickness have been shown to be similar suggesting that the increased permeability of scalp skin is due increased absorption via the follicular route, for which hydrophilic drug molecules show greater increases in permebility (11,35). These different skin characteristics, where the predominance of a particular permeation mechanism may be altered guard against automatically correlating findings produced with skin from different anatomical areas.

#### Finite Dose In Vitro Skin Permeation and Distribution Studies over 24 H

In vitro finite dose permeation studies were conducted over 24 h to more closely mimic in use conditions in comparison to infinite dose experiments. In addition, the influence of short periods of externally applied heat, such as are likely to be feasible to use in a clinical scenario were investigated for their effects on drug deposition into the hair follicles, different skin layers and permeation through human scalp skin. The short durations of heat (~ 43°C, ca. 20 and 30 min) were produced using sodium thiosulfate (ST) solution as previously described by Farah et al. (2019) (15) and comparisons with no ‘additional’ heat (32°C) and water bath heating (45°C) were made. The potential of phase change materials such as ST as well as devices for generating localised heating to improve dermal absorption have been highlighted elsewhere (36–38). It is likely that such devices could be used in the home by patients following appropriate training in the same way as heating patches for musculoskeletal pain or hand warming pouches containing sodium acetate are commercially available for individuals’ use (38). In the clinical situation increasing the skin’s temperature would be expected to increase cutaneous blood flow as well as increasing drug delivery into and across the skin (39). This increased blood flow may increase drug clearance from the skin tissue, increase systemic exposure and negate the impact of improving drug delivery across the skin. However previous studies with the commercialised product Rapydan which successfully used heat to increase drug delivery to the skin and clinically improved cutaneous anaesthesia suggests that any increased drug clearance will not negate the impact on increased drug delivery (40). Moreover any increased systemic exposure of the drug from a heated topical system is still likely to be considerably lower than if the drug was administered via oral administration. Finasteride was delivered from filtered saturated solutions in 1:1 binary solvent system (IPA:PG and IPA:TP) to investigate whether synergy in penetration enhancement occurred when mixed solvent systems were used. IPA was used in both mixed solvent vehicles as it provided the highest delivery at the higher temperature from an infinite dose. TP and PG also provided good delivery of finasteride to the skin and enhancement with heat and were selected for inclusion as co-solvents with IPA in the 1:1 prototype binary solvent systems.

As finasteride showed temperature dependent solubility, filtered saturated solutions of the drug were prepared at both 32°C and 45°C and used in the unheated and heated experiments respectively. This enabled the effects of heat on drug delivery be assessed separately from that of heat on the thermodynamic activity of finasteride in the vehicles. This provided an applied dose of 1629.6 μg and 900.7 μg for IPA:PG and IPA:TP respectively at 32°C and 1727.6 μg and 1038.9 μg at 45°C.

**Fig. 3:**
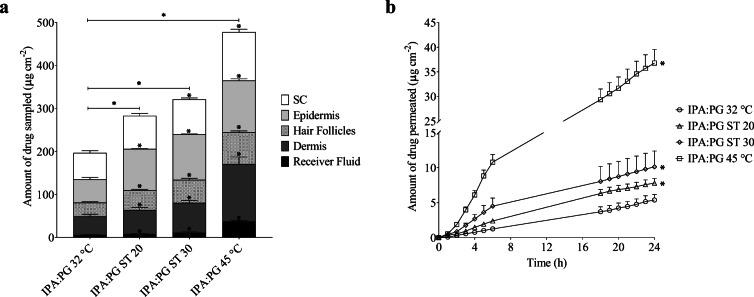
(**a**) The amount of finasteride recovered from the stratum corneum (SC), epidermis, hair follicles, dermis and receiver fluid at the end of the 24 h finite dose permeation study from IPA:PG (1:1) binary system; (**b**) mean cumulative amount of finasteride permeated through human scalp skin over 24 h from IPA:PG (1:1) binary system. Experiments were conducted at 32°C with and without ST solution producing heat (~ 43°C) for Ca. 20 & 30 mins (ST 20 & ST 30 respectively) and at 45°C (without ST). All bars are mean + SEM of n = 6 diffusion cells. * denotes significant difference in the amount of drug recovered compared to the control (32°C, no additional heat)

The results for finasteride recovery from the hair follicles and the different skin layers (SC, epidermis and dermis) following the differential tape stripping and the permeation profiles for the IPA:PG vehicle are shown in Fig. 3 (a) and (b) respectively. In terms of the total amount of drug delivered (SC + hair follicles+ epidermis + dermis + receiver fluid), all of the heating conditions significantly increased the amount of finasteride delivered, with ST 20 and ST 30 increasing the delivery 1.44 and 1.63 fold respectively in comparison to the experiment at 32°C. For comparative purposes water bath heating was also used for the whole 24 h (IPA:PG 45°C), which provided highest delivery overall, 2.43 fold greater than (IPA:PG 32°C). At each of the heating conditions the amount of finasteride delivered to each skin layer was significantly increased in comparison to (IPA:PG 32°C) with the exception of the stratum corneum with IPA:PG ST 20. Specifically considering the hair follicles, the application of heat led to a significant increase in the amount of finasteride recovered, with IPA:PG ST 20 and IPA:PG ST 30 providing 1.44 and 1.69 fold increases respectively. As with total delivery, the highest amounts of drug were obtained when the skin was heated for the entire 24 h period, IPA:PG 45°C. All the IPA:PG permeation profiles follow typical finite dose profiles as can be seen in Fig. 3 (b). The effect of heat on permeation across the skin was apparent from early time points, for example after 2 h delivery from IPA:PG ST 30 was significantly higher than IPA:PG ST 20 and IPA:PG 32°C. With water bath heating, delivery was significantly increased over all other conditions from 2 h onwards. After 24 h the enhancement with heat ranged from 1.45 to 1.88 fold for IPA:PG ST 20 and IPA:PG ST 30 respectively and 6.83 fold with IPA:PG 45°C.

**Fig. 4:**
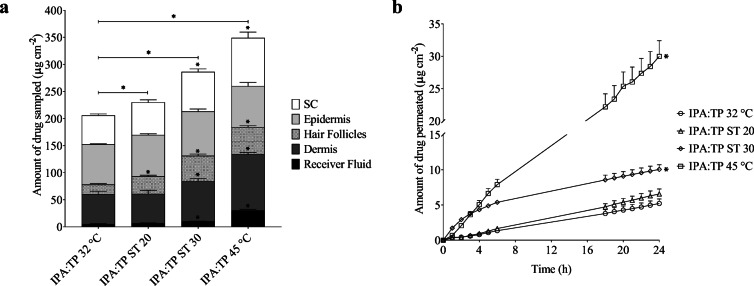
(**a**) The amount of finasteride recovered from the stratum corneum (SC), epidermis, hair follicles, dermis and receiver fluid at the end of the 24 h finite dose permeation study from IPA:TP (1:1) binary system; (**b**) mean cumulative amount of finasteride permeated through human scalp skin over 24 h from IPA:TP (1:1) binary system. Experiments were conducted at 32°C with and without ST solution producing heat (~ 43°C) for Ca. 20 & 30 mins (ST 20 & ST 30 respectively) and at 45°C (without ST). All bars are mean + SEM of *n* = 6 diffusion cells. * denotes significant difference in the amount of drug recovered compared to the control (32°C, no additional heat)

The results for IPA:TP binary system are shown in Fig. 4 (a) and (b). With respect to the total amount of drug delivered (SC + hair follicles+ epidermis + dermis + receiver fluid), all of the heating conditions significantly increased the amount of finasteride delivered, with ST 20 and ST 30 increasing the delivery 1.12 and 1.39 fold respectively in comparison to the experiment at 32°C. Again water bath heating for the whole 24 h (IPA:TP 45°C) provided the highest finasteride delivery, 1.70 fold greater than (IPA:TP 32°C).

The application of heat led to a significant increase in the amount of finasteride recovered from the hair follicles, with IPA:TP ST 20 and IPA:TP ST 30 providing 1.79 and 2.58 fold increases respectively. When considering the delivery of finasteride to the deeper skin layers, only the increase in delivery to the dermis and receiver fluid with IPA:TP ST 30 and IPA:TP 45°C were statistically significant. This is suggestive that heat is facilitates delivery via the follicular route to the deeper skin layers and receiver fluid rather than through increased transport through the continuous stratum corneum.

The finasteride permeation profiles from the IPA:TP vehicle can be seen in Fig. 4 (b). Over the first 2 h of the permeation study, ST 30 delivered significantly more drug into the receiver fluid compared to all the other heating conditions investigated, exhibiting a very short lag time. With the other conditions a more typical finite dose profile with an initial lag phase was observed. Continuous water bath heating significantly increased finasteride delivery over ST 30 from 4 h onwards. After 24 h significant 1.93 fold enhancement of finasteride permeation was observed with heat from IPA:TP ST 30, in contrast significant enhancement was not observed with IPA:TP ST 20. This behaviour is more complex than what was observed with IPA: PG which provided enhancements with both ST 20 and ST 30 in a predictable fashion suggesting that the amount of finasteride delivered into the receiver fluid from IPA:PG can be tailored by controlling the length of time external heat is applied to the skin. For IPA:TP potentially a greater effect may be obtainable, but the heat has to be used for the longer duration to achieve this. The rapid delivery of finasteride at early time points with IPA:TP ST 30 was seen previously with isotretinoin in a different mixed solvent system and is suggestive of follicular delivery of the drug which is known to be rapid (12,15,41,42). The total amount of delivery into the skin from IPA:PG was greater than that from IPA:TP. This is likely to be related to the higher dose applied as filtered saturated solutions were used to dose the Franz cells and the solubility of finasteride in IPA:PG was higher than in IPA:TP. This is supported by consideration of the percentage of the applied dose recovered from each skin compartment, receiver fluid and total recovery which is shown in Table 3. These data demonstrate that the percentage of the applied dose delivered from IPA:TP was actually greater than that from than IPA:PG, even though IPA:PG delivered the largest amount of drug overall. These data also show that a higher percentage of the applied dose was delivered in the presence of heat and therefore confirms that the higher dose applied with the heated systems does not itself explain the improved delivery of the drug when heat is applied. Significantly the finite dose experiments over 24 h from both IPA:TP and IPA:PG confirm that improved delivery from short durations of heat is still apparent after 24 h.

**Table 3:** Percentage of finasteride recovered from hair follicles, stratum corneum (SC), epidermis, dermis and receiver fluid in the 24 h finite dose study (Mean ± SEM, n = 6)

Vehicle	Treatment condition	Hair follicles	SC	Epidermis	Dermis	Receiver fluid	Total recovery
IPA:PG	32°C	1.95 ± 0.13	3.80 ± 0.32	3.32± 5.59	2.67 ± 0.32	0.25 ± 0.06	94.91 ± 3.69
	ST 20	2.66 ± 0.13	4.45 ± 0.34	5.59 ± 0.07	3.23 ± 0.35	0.35 ± 0.07	93.96 ± 2.73
	ST 30	3.11 ± 0.21	4.72 ± 0.20	6.11 ± 0.08	4.02 ± 0.33	0.56 ± 0.15	93.45 ± 2.49
	45 °C	4.27 ± 0.20	6.54 ± 0.38	6.98 ± 0.22	7.74 ± 0.96	1.86 ± 0.39	92.85 ± 3.45
IPA:TP	32°C	2.01 ± 0.21	5.94 ± 0.31	8.23 ± 0.13	6.08 ± 0.64	0.58 ± 0.07	95.34 ± 3.70
	ST 20	3.11 ± 0.23	5.79 ± 0.46	7.37 ± 0.19	5.23 ± 0.61	0.63 ± 0.07	90.94 ± 3.00
	ST 30	4.48 ± 0.31	7.05 ± 0.50	7.89 ± 0.43	7.16 ± 0.43	0.97 ± 0.06	92.53 ± 3.15
	45 °C	4.85 ± 0.25	8.58 ± 1.05	7.27 ± 0.66	10.00 ± 0.32	2.88 ± 0.23	96.30 ± 4.20

#### Finite Dose In Vitro Skin Permeation and Distribution Studies after 1 H

**Fig. 5:**
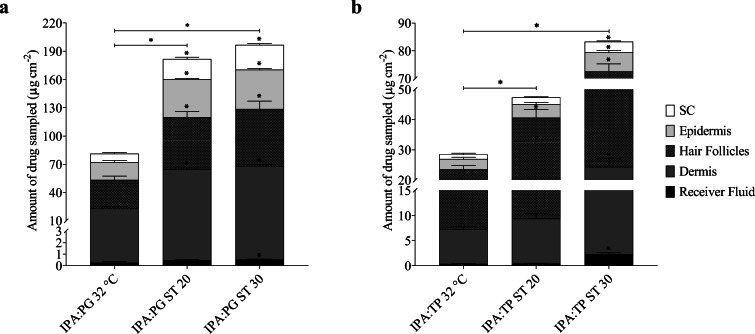
Distribution of finasteride across human scalp skin and receiver fluid at the end of a 1 h permeation study from (**a**) IPA:PG (1:1) binary system and (**b**) IPA:TP (1:1) binary system. Experiments were conducted at 32°C with and without ST solution producing heat (~ 43°C) for Ca. 20 & 30 mins (ST 20 & ST 30 respectively). All bars are mean + SEM of *n* = 4 diffusion cells. * denotes significant difference in the amount of drug recovered compared to the control (32°C, no additional heat)

In order to better understand the drug transport mechanism in response to heat, the finite dose experiments were repeated and stopped after 1 h. This enabled the distribution of finasteride in the different skin compartments to de determined at a time point soon after the application of the externally applied heat. These results are presented in Fig. 5 (a) and (b) for IPA:PG and IPA:TP respectively and Table 4 shows these data as the percentage of drug recovered at the end of the 1 h experiment.

**Table 4:** Percentage of finasteride recovered from hair follicles, stratum corneum (SC), epidermis, dermis and receiver fluid in the 1 h finite dose study (Mean ± SEM, n = 4)

Vehicle	Treatment condition	Hair follicles	SC	Epidermis	Dermis	Receiver fluid	Total recovery
IPA:PG	32°C	1.87 ± 0.20	0.55 ± 0.06	1.15 ± 0.09	1.40 ± 0.08	0.02 ± 0.004	94.86 ± 2.62
	ST 20	3.20 ± 0.29	1.25 ± 0.09	2.34 ± 0.06	3.71 ± 0.07	0.03 ± 0.004	94.45 ± 2.51
	ST 30	3.49 ± 0.41	1.52 ± 0.07	2.43 ± 0.06	3.92 ± 0.06	0.03 ± 0.002	96.57 ± 1.23
IPA:TP	32 °C	1.81 ± 0.12	0.18 ± 0.03	0.37 ± 0.06	0.77 ± 0.04	0.04 ± 0.002	97.03 ± 1.59
	ST 20	3.01 ± 0.21	0.21 ± 0.02	0.43 ± 0.05	0.86 ± 0.07	0.04 ± 0.003	97.89 ± 2.91
	ST 30	4.63 ± 0.22	0.36 ± 0.02	0.67 ± 0.05	2.13 ± 0.21	0.22 ± 0.03	96.52 ± 3.66

The total amount of finasteride recovered (from the SC, epidermis, dermis, hair follicles and receiver fluid) was significantly enhanced with the ST heating systems for both IPA:PG and IPA:TP binary systems. With ST 20 the level of enhancement ranged from 1.66 to 2.24 fold for IPA:TP and IPA:PG respectively. Greater levels of enhancement were obtained with ST 30 ranging from 2.42 to 2.92 fold for IPA:PG and IPA:TP respectively.

The amount of finasteride recovered from the hair follicles after 1 h for IPA:PG 32°C and IPA:TP 32°C were equal to the amounts recovered after 24 h under the same conditions, supporting the hypothesis that transport into and through the hair follicles is a relatively rapid process (12,15,41–43). When either heating system (ST 20 or ST 30) was used, the amounts of finasteride recovered from the hair follicles were increased over the 32°C experiments and the respective experiments conducted over 24 h with ST 20 or ST 30. In agreement with previous work this again provides strong evidence that heat increases follicular drug delivery (14,15). In these previous studies, heat focussed delivery via the hair follicles which was apparent through larger increases in the hair follicle drug content and that of the dermis and receiver fluid in comparison to the stratum corneum and epidermis. Here, the effect of heat on the permeation of finasteride across scalp skin was specific to the vehicle composition. For example the increase in the amount of finasteride being recovered from the SC (as a percentage of the applied dose – Table 4) was larger than that of the hair follicles for the heated IPA:PG experiments whereas with IPA:TP the increase in the hair follicle finasteride content with heat was larger than that of the SC or epidermis suggesting that that formulation vehicle focussed finasteride delivery towards the hair follicles. Our previous work similarly found that the selection of penetration enhancer affected follicular delivery of drugs in the presence of heat (14,15). The relative contribution of follicular transport to lipophilic molecules with a steroidal structure such as finasteride has been determined to be relatively low in comparison to more hydrophilic molecules, suggesting that particular enhancers may be required to favour its delivery via this route (44). TP has been shown previously to interact with model sebum to a greater extent than PG possibly influencing its ability to affect follicular delivery (45). The data in Table 4 confirms that the IPA:TP ST30 formulation delivered a larger percentage of the applied dose to the hair follicles and a considerably lower dose to the stratum corneum indicating that selection of a specific combination of formulation vehicle and heating duration can be used to focus drug delivery to the hair follicles if this is desired. This may be preferable than simply maximising the dose of finasteride that can be delivered topically for example with the IPA:PG ST30 system. Previously, limiting topical doses of finasteride was found to be necessary to enable scalp dihydrotestosterone levels to be suitably lowered whilst minimising changes to serum dihydrotestosterone levels which are associated with systemic side effects (46).

## Conclusions

The use of short durations of heat in combination with chemical penetration enhancers was able to increase the delivery of finasteride to human scalp skin including to the hair follicles. The chemical penetration enhancers selected affected the extent to which heat improved finasteride delivery to the skin and the distribution of the drug within the skin, with particular combinations of penetration enhancers focussing drug delivery via the hair follicles. The data support the concept that short durations of externally applied heat may be used to improve topical delivery of drugs to the skin including when delivery to the hair follicles is specifically desired.

### Acknowledgments and Disclosures

The authors would like to thank MedPharm Ltd. (UK) for funding this work.
